# Chromosome Stability of Synthetic-Natural Wheat Hybrids

**DOI:** 10.3389/fpls.2021.654382

**Published:** 2021-03-17

**Authors:** Laibin Zhao, Die Xie, Chaolan Fan, Shujie Zhang, Lei Huang, Shunzong Ning, Bo Jiang, Lianquan Zhang, Zhongwei Yuan, Dengcai Liu, Ming Hao

**Affiliations:** ^1^Triticeae Research Institute, Sichuan Agricultural University, Chengdu, China; ^2^State Key Laboratory of Crop Gene Exploration and Utilization in Southwest China, Sichuan Agricultural University, Chengdu, China; ^3^Wheat Center, Henan Institute of Science and Technology, Xinxiang, China

**Keywords:** chromosome instability, aneuploidy, polyploidization, synthetic hexaploid wheat, meiosis

## Abstract

Primary allopolyploids are not only ideal materials to study species evolution, but also important bridges in incorporating genetic diversity of wild species into crops. Primary allopolyploids typically exhibit chromosome instability that a disadvantage trait in crop breeding. Newly synthesized hexaploid wheat has been widely used in wheat genetics and breeding studies. To better understand the cytological and genetic basis of chromosome instability, this study investigated the chromosomes of a large number of seeds derived from the synthetic wheat SHW-L1 and its hybrids with natural wheat. SHW-L1 exhibited persistent chromosome instability since we observed a high frequent chromosome variation *de novo* generated from euploid SHW-L1 plants at the 14th generation of selfing (F_14_). High frequent chromosome variations were also observed in the F_2_ hybrids and most of the analyzed recombinant inbred lines (RILs) at F_14_, derived from the cross of SHW-L1 with common wheat variety Chuanmai 32. Chromosome instability was associated with frequent univalency during meiotic metaphase I. The experiment on reciprocal crosses between SHW-L1 and Chuanmai 32 indicated that cytoplasm has not obvious effects on chromosome instability. An analysis on 48 F_14_ RILs revealed chromosome variation frequency was not associated with the *Ph1* alleles from either SHW-L1 or Chuanmai 32, rejecting the hypothesis that chromosome instability was due to the *Ph1* role of synthetic wheat. In the analyzed RILs, chromosome instability influences the phenotype uniformity, showing as obvious trait differences among plants within a RIL. However, the analyzed commercial varieties only containing ∼12.5% genomic components of synthetic wheat were chromosomally stable, indicating that chromosome instability caused by synthetic wheat can be effectively overcome by increasing the genetic background of common wheat.

## Introduction

Allopolyploids are common in natural ecosystems (Grant, 1981). They arise as a result of distant hybridization (and chromosome doubling) between related species containing different sets of related, but not completely homologous, chromosomes known as homoeologues. Many important crop species, such as bread and durum wheat, oat, cotton, sugarcane, canola, coffee, and tobacco are allopolyploids and have become genetically isolated from their progenitor species. Because of evolution and domestication bottlenecks (Stebbins, 1950; Buckler et al., 2001), allopolyploids usually evolved from a small number of founder amphiploids, thereby excluding much of the genetic variation harbored by the progenitor species. The excluded variation can be recaptured by using artificially re-synthesized allopolyploids that duplicate the allopolyploidization process (Kihara, 1944; McFadden and Sears, 1944).

Primary allopolyploids (neoallopolyploids) often exhibit a high degree of genetic instability (Ramsey and Schemske, 2002; Comai, 2005; Madlung et al., 2005; Feldman and Levy, 2012; Li et al., 2016), globally envisaged as a consequence of genomic shock (McClintock, 1984). Studies on divergent plant taxa have demonstrated that chromosome instability is common in primary allopolyploids (Gupta and Priyadarshan, 1982; Ramsey and Schemske, 2002; Zhou, 2003; Gaeta et al., 2007; Lim et al., 2008; Mestiri et al., 2010; Xiong et al., 2011; Chester et al., 2012; Zhang et al., 2013). Chromosome instability results in a high frequency of aneuploidy characterized by changes in copy number of entire chromosomes or parts relative to the standard karyotype (means the standard chromosome complement). This can pose a problem for plant breeders in that the achievement of regulated varietal uniformity might not be possible. High frequencies of aneuploids, which can be seen as off-types in the field, can be a problem (Worland and Law, 1985). Chromosome instability is hence a disadvantage trait in breeding.

Bread wheat (*Triticum aestivum* L. AABBDD, 2*n* = 6*x* = 42) is an allohexaploid that arose from a polyploidization event involving tetraploid *T*riticum *turgidum* (2*n* = 4*x* = 28, AABB) and diploid *Aegilops tauschii* (2*n* = 2*x* = 14, DD) (Kihara, 1944; McFadden and Sears, 1944). Modern wheat varieties are considered to be chromosomally stable but can produce 1–3% of aneuploid individuals (Riley and Kimber, 1961). However, chromosomal variation is ubiquitous in some newly developed synthetic hexaploid wheat (SHW) created by crossing *T. turgidum* with *A. tauschii* (Mestiri et al., 2010; Zhang et al., 2010). In a systematical study on 16 SHW lines, the frequencies of aneuploidy varied from 20–100% among plants in each line (Zhang et al., 2013). Despite this, SHWs and their derived populations have been widely used in genetic studies (Mujeeb-Kazi et al., 1996; Sorrells et al., 2011; Hao et al., 2019) and the assembling of genome sequences (Chapman et al., 2015). SHWs have been used as donor parents to widen the genetic base of bread wheat by numerous groups. Many studies have confirmed that SHWs can enhance yield and other traits across a diverse range of environments (Hoisington et al., 1999; Warburton et al., 2006; Dreisigacker et al., 2008; Trethowan and Mujeeb-Kazi, 2008; Yang et al., 2009; Ogbonnaya et al., 2013; Borner et al., 2015; Hao et al., 2019).

However, chromosome instability in SHWs has attracted little attention compared to other breeding traits with previous investigations limited to studies chromosome numbers. Some genetic questions on chromosome instability remain to be answered. For example, what are the underlying cytological and genetic mechanisms and how such effects can be overcome in genetic and breeding studies? In this study, we used the synthetic wheat accession SHW-L1 and hybrids with current wheat genotypes to systematically observe chromosome variation, examine the role of cytoplasm in reciprocal hybrids, and evaluate the effect of *Ph1* (*Pairing homoeologous 1*) on chromosome stability. Although the present results did not offer clear answers, they provide new information in regard to adverse effects for genetic and breeding studies.

## Materials and Methods

### Plant Materials

The materials subjected to analysis were the synthetic hexaploid SHW-L1, its parents Chinese *T*. *turgidum* L. ssp. *turgidum* landrace AS2255 (female) and Iranian *A*. *tauschii* ssp. *tauschii* accession AS60 (Zhang et al., 2004), commercial cultivar Chuanmai 32 (CM32), F_1_ and F_2_ populations of reciprocal crosses between SHW-L1 and CM32, as well as a set of F_14_ recombinant inbred lines (RILs) derived from cross SHW-L1 × CM32. SHW-L1 was produced in 2001 (Zhang et al., 2004). In 2002, a SHW-L1 plant (the first generation of the synthetic hexaploid, S_1_) with 21 pairs of chromosomes was used as female in a cross with CM32. One hybrid plant was used to develop the F_14_ RILs and the SHW-L1 S_1_ plant was selfed to S_14_. The SHW-L1/CM32 RIL population was genotyped using wheat 660K SNP array (Yang, 2016; Zhao et al., 2020). SHW-L1-derived commercial varieties Shumai 969 and Shumai 830 were used to determine aneuploid frequencies. These two varieties contained ∼12.5% of SHW-L1 genome, derived from a double top-cross breeding program using three common wheat varieties (Hao et al., 2019).

### Evaluation of Agronomic Traits

Phenotypic data were obtained from a previous experiment involving the SHW-L1/CM32 RIL population (Hao et al., 2019). All materials were space-planted in 2-m row, with 30 cm between rows at Wenjiang experimental station (30°36′N, 103°41′W) (2016, 2017) and Beijing (2017) with three replications at each site. Traits including spike length (Wenjiang 2016, Wenjiang 2017, and Beijing 2017), spikelet number (Wenjiang 2016, Wenjiang 2017, and Beijing 2017), uppermost internode length (Beijing 2017), and plant height (Beijing 2017) were evaluated at maturity from the tallest tiller of 10 randomly selected plants in each line. The numbers of seeds in the first and second florets of each spikelet were scored to calculate the seed-setting rate (Beijing 2017), and 20 spikes were used for each line. The standard deviation for each trait among different individuals from each RIL was then calculated.

### Cytological Observations

Chromosome numbers in root-tip cells and at meiotic metaphase I in pollen mother cells (PMC) were determined as described by Zhang et al. (2007). Chromosome numbers were determined in eight seedlings from each of five plants in each of 48 RILs. More seedlings from five lines (L9, L17, L25, L36, and L82) were scored. Chromosome number was determined if at least four root-tip cells had the same chromosome number. Weighted mean aneuploid frequency from five plants was used as an index of each line. One seedling from each of the 48 RILs was used to analyze chromosome constitution by *in situ* hybridization.

Slides were prepared for fluorescence *in situ* hybridization (FISH) and genomic *in situ* hybridization (GISH) as previously described (Hao et al., 2011; Zhao et al., 2016). Probes included in the hybridization solution were 6-carboxyfluorescein (6-FAM) or 6-carboxytetramethylrhodamine (Tamra) labeled oligo-pTa-535, oligo-pSc119.2 (Tang et al., 2014), oligo-pTa71, and (CTT)_10_ (Pickering et al., 2006) synthesized by Sangon Biotech Co., Ltd. (Shanghai). Genomic DNA for GISH was isolated from young leaves of PI428205 (*Triticum urartu*), PI330488 (*Ae*gilops *speltoides*), and AS2388 (*A. tauschii*), by a modified CTAB method (Kidwell and Osborn, 1992). Genomic DNA of *T. urartu* and *A. tauschii* was labeled by nick translation with Chroma Tide Alexa Fluor 488-5-dUTP (Invitrogen, United States; no. C11397, green coloration) and Texas Red-5-dCTP (Perkin-Elmer, United States; no. NEL 426001EA, red coloration), respectively. Genomic DNA of *A. speltoides* was used as a blocker. The latter steps, including hybridization, image gathering, image processing and re-hybridization were described in Zhao et al. (2018).

### Statistical Analyses

Statistics was performed using the data analysis function in Microsoft Excel and SPASS statistical software, version 22 (IBM SPASS).

## Results

### *De novo* Production of Chromosome Variations in Synthetic Wheat

**FIGURE 1:**
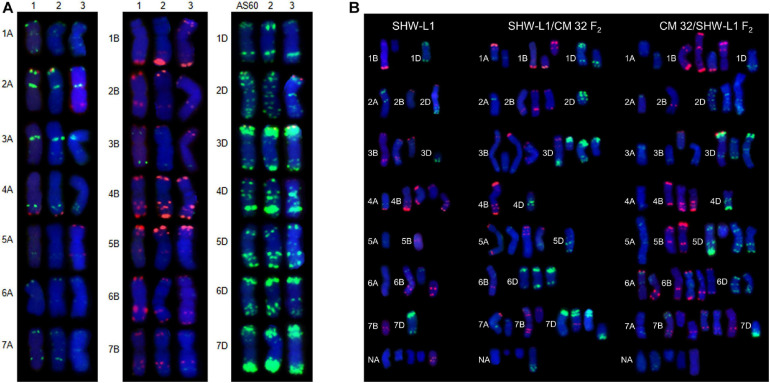
FISH karyotypes. **(A)** FISH karyotypes of SHW-L1 with its parents and Chuanmai 32. 1, tetraploid wheat AS2255; 2, SHW-L1; 3, Chuanmai 32. **(B)** Chromosomal structural variations detected in SHW-L1 and F_2_ hybrids derived from reciprocal crosses between SHW-L1 and Chuanmai 32. Green FISH signals are from probe oligo-pTa-535; red signals are from probe oligo-pSc119.2.

Chromosome numbers were made on CM32, SHW-L1 and their hybrids (Table 1). We considered a seedling as aneuploid if its chromosome number was not 42. Common wheat variety CM32 generated rare aneuploidy (1.3%) consistent with a previous study (Riley and Kimber, 1961). However, SHW-L1 generated high frequency of aneuploids. Seeds were harvested from 13 euploid SHW-L1 plants with 21 pairs of chromosomes confirmed by FISH (Figure 1A). Among 181 seedlings, 88 (48.6%) were aneuploid (Supplementary Table 1). Chromosome numbers ranged from 39 to 45 (Table 1). FISH was then used to identify chromosome constitutions. Among 70 seedlings with 2*n* = 42, 10 (14.3%) were not true euploids (called as “hidden aneuploids” by Zhang et al., 2013) in that they did not have 21 pairs of chromosomes (Supplementary Table 1). These aneuploids were *de novo* generated from euploid plants, indicating that SHW-L1 was cytologically unstable.

**TABLE 1:** Chromosome variations in synthetic wheat SHW-L1 and its derivatives.

Plant material	Number Of plants or lines	Number Of observed seeds	% for 2*n* ≠ 42 (mean, range)	Number distribution*	Structural variation %**
SHW-L1	13	181	48.6 (51.9, 16.7–83.3)	39–45	20.4
SHW-L1/CM 32 F_1_	3	95	68.4 (68.0, 66.7–69.4)	38.5–46	35.5
CM 32/SHW-L1 F_1_	3	85	64.7 (64.3, 60.0–68.4)	39.5–44	39.0
RILs F_14_	48	1437	24.6 (22.8, 0.0–92.5)	38–49	NA
Chuanmai 32	5	78	1.3	41–42	NA
Shumai 969	Bulked	74	1.3	42–42.5	NA
Shumai 830	Bulked	100	3.0	41–42	NA

Bulked, the seed were harvested together regardless of individual; NA, data was unavailable. *0.5 represented a chromosome fragment. **Structural variation was according to FISH.

Fluorescence *in situ* hybridization karyotypes of SHW-L1 were compared to those of the *T. turgidum* and *A. tauschii* parents (Figure 1A) to identify structural variations. Among 137 SHW-L1 seedlings derived from euploid plants, 28 (20.4%) had unambiguous structural variations involving 39 chromosomes (Figure 1B and Supplementary Table 2). Most (21) had lost one chromosome or chromosome fragment. Ten had altered FISH signals or chromosome translocations (including one dicentric translocation, three Robertsonian translocations, and one small terminal translocation). These results suggested that breakage and breakage-fusion were the main causes of chromosomal variation. Of the above 39 variant chromosomes, 21 (53.8%) were from B genome, four (10.3%) from A genome, and four from D genome, and ten (25.6%) were undetermined (Supplementary Table 3).

### Effects of Cytoplasm and Hybridization on Chromosome Variations

Six F_1_ plants from the reciprocal cross between SHW-L1 and CM32 with 21 pairs (Supplementary Figure 1) were selfed. The reciprocal crosses had similar proportions of F_2_ seedlings with 2*n* ≠ 42 (68.0 vs 64.3%; *t*-test, *p* = 0.23) (Table 1), indicating that cytoplasm had no effect on the frequency of aneuploidy. Among 180 F_2_ seedlings from the reciprocal cross, 66.7% (120) had 2*n* ≠ 42, higher than the frequency observed in the SHW-L1 parent (48.6%) (Table 1) (*t*-test, *p* = 0.03). Among these plants, 175 were examined by FISH and similarly, higher frequencies of structural variations were present among F_2_ seedlings of SHW-L1/CM32 (35.5%, 33/93) and CM32/SHW-L1 (39.0%, 32/82) compared to that in SHW-L1 (20.4%) (Table 1 and Supplementary Table 1). This indicated that the hybridization led to higher levels of cytological instability. FISH-revealed chromosomal structure between the newly synthesized wheat SHW-L1 and modern variety CM32 has obvious differences (Figure 1A), and it was likely that chromosome heterozygosity in their hybrids led to the increased meiotic instability.

In both SHW-L1 (A:B:D = 4:21:4) and its hybrids chromosome structural variations were biased toward the B genome (SHW-L1/CM32, A:B:D = 9:30:20; CM32/SHW-L1, A:B:D = 11:33:17) (Supplementary Table 3). Although the hybrids had higher variations for all three subgenomes, the frequencies involving the D genome increased somewhat compared to SHW-L1, suggesting a higher chromosome differentiation in D genome between SHW-L1 and CM32.

### Chromosome Variation in Synthetic-Derived Lines

Chromosome number variation in 48 F_14_ SHW-L1/CM32 RILs was then analyzed. Since FISH data was not obtained the frequencies of “hidden aneuploids” (2*n* = 42) were undetermined and all plants with 2*n* = 42 were treated as euploids. Compared to SHW-L1 and F_2_ hybrids, chromosome instability was lower in the RILs (Table 1). Among 1,437 seedlings, 1,083 (75.4%) had 2*n* = 42. Chromosome numbers among the others varied from 37 to 49 (Supplementary Table 4). The frequencies of progeny with 2*n* = 42 among all 48 RILs were skewed toward high levels of euploidy (Shapiro–Wilk test, *p* < 0.01) (Supplementary Table 4). Of them, six (12.5%) RILs showed no aneuploid. We also observed some intergenomic chromosomal translocations (Supplementary Figure 2) and chromosome fragments (Supplementary Table 4).

The commercial derivatives Shumai 969 and Shumai 830 selected from second generation crosses of SHW-L1 with common wheat genotypes (Hao et al., 2019) were chromosomally stable, exhibiting 1.3 and 3.0% aneuploids, respectively (Table 1).

### Abnormal Meiosis in the Synthetic and Its Hybrids

**FIGURE 2:**
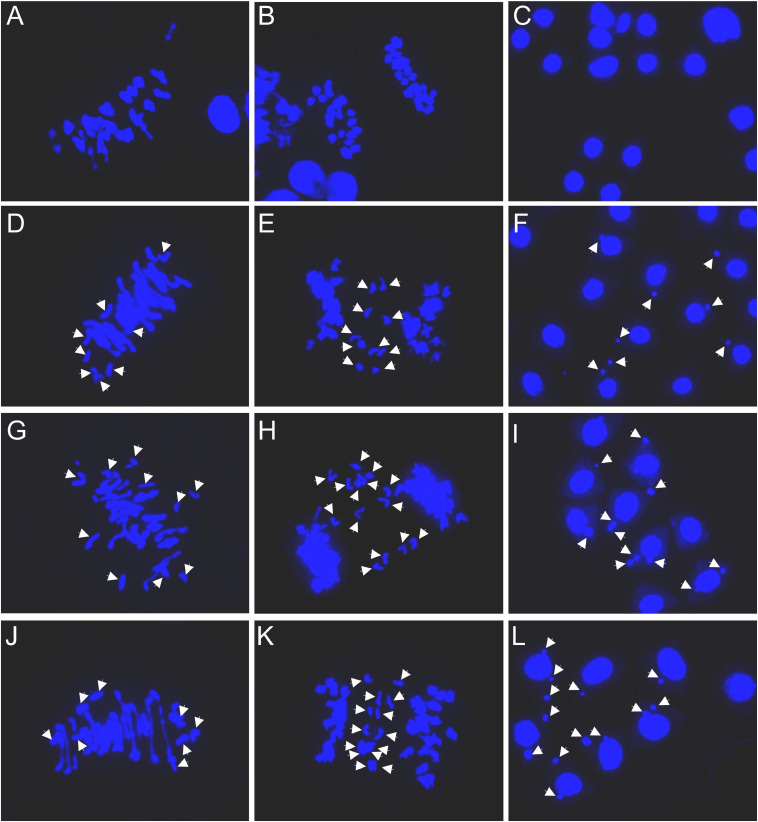
Meiotic observations: **(A–C)** Chuanmai 32; **(D–F)** SHW-L1; **(G–I)** SHW-L1/Chuanmai 32 F_1_; **(J–L)** Chuanmai 32/SHW-L1 F_1_. **(A,D,G,J)** are metaphase I. **(B)**; **(E,H,K)** are at ana-/telophase I; **(C,F,I,L)** are tetrads. White arrows indicate univalents **(D,G,J)**, separation of chromatids in first division **(E,H,K)**, and micronuclei **(F,I,L)**.

Common wheat variety CM32 showed normal meiosis (Figures 2A–C). In 39 cells observed only two had univalents (Table 2). However, univalents were present at metaphase I in most of the 2*n* = 42 plants of SHW-L1 (Figure 2D). There was a mean 5.23 univalents per cell in 65 cells (Table 2). F_1_ hybrids with CM32 had higher numbers of univalents (Figures 2G,J); 8.21 for SHW-L1/CM32 and 7.12 for CM32/SHW-L1 (*p* = 0.0001 and 0.004, *t*-test), respectively. However, there was no difference in univalent number between the two hybrids (*p* = 0.10, *t*-test). The univalents had a tendency to divide mitotically as laggards in anaphase/telophase I (Figures 2E,H,K). Micronuclei were also common at the tetrad stage (Figures 2F,I,L). In summary, compared to CM32, SHW-L1 and its F_1_ hybrids had significant numbers of unpaired chromosomes favoring the production of chromosome variations.

**TABLE 2:** Meiotic features in euploid plants of synthetic SHW-L1 and its hybrids.

Plant materials	Univalents			Laggards		Micronuclei	
	Number of cells	Number of cells with univalent (%)	Number of univalents per cell	Number of cells	Number of cells with laggards (%)	Number of cells	Number of cells having micronuclei (%)
Chuanmai 32 (2*n* = 42)	39	2 (5.1)	0.10	16	0	43	0
SHW-L1 (2*n* = 42)	65	58 (89.2)	5.23	50	44 (88.0)	104	79 (76.0)
SHW-L1/Chuanmai 32 F_1_ (2*n* = 42)	58	58 (100.0)	8.21	79	73 (92.4)	40	30 (75.0)
Chuanmai 32/SHW-L1 F_1_ (2*n* = 42)	73	72 (98.6)	7.12	57	52 (91.2)	42	39 (92.9)

**FIGURE 3:**
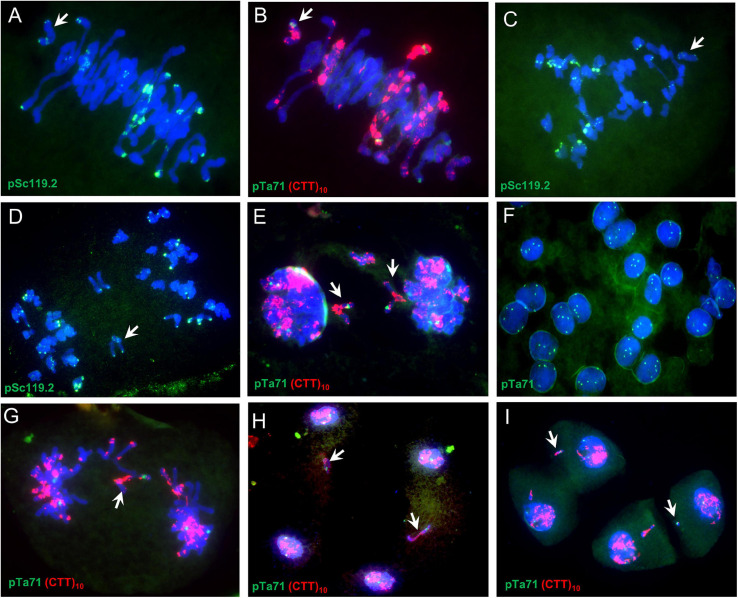
Meiosis in a SHW-L1 monosomic 6B plant. **(A,B)** Metaphase I; **(C)** Anaphase I; **(D,E)** Ana-/telophase I; **(F)** Dyads, signals showing secondary constrictions of chromosome 1B, 6B, and 5D; **(G,H)**; Anaphase II to telophase II; **(I)** Tetrads. White arrows indicate chromosome 6B or fragments thereof.

The univalent behaviors were further detected by using a monosomic 6B SHW-L1 plant (Figure 3). FISH analysis indicated that examined univalent 6B chromosome (37/37; present in all 37 PMCs) (Figures 3A,B) lagged (5/5) (Figure 3C) and divided at anaphase I (47/48) (Figures 3D,E), and then the divided 6B went into each dyad (68/71) (Figure 3F). The 6B chromosome then remained on the equatorial plate during anaphase II (36/38) (Figures 3G,H), forming a micronucleus separated from the resulting nucleus (58/84) (Figure 3I).

### Aneuploidy Was Not Related to the *Ph1* Locus

*Ph1* is a major gene that restricts chromosome pairing to homologs in both tetraploid and hexaploid wheat. *TaZIP4-B2* (*TraesCS5B02G255100*) was shown to be a candidate gene for *Ph1* (Rey et al., 2017). To test whether the abnormal meiosis of the synthetic wheat SHW-L1 and its hybrids was related with the *Ph1* (inherited from its tetraploid wheat parent AS2255), we re-analyzed the 660 K SNP genotyping data of SHW-L1/CM32 RILs (Yang, 2016; Zhao et al., 2020). According to the SNP alleles distribution on chromosomes, 21 out of the 48 analyzed RILs may inherit the *Ph1* gene from CM32 and they had an average aneuploid frequency of 29.2%; the other 27 RILs may inherit *Ph1* gene from SHW-L1 and they had an average of aneuploid frequency of 17.7% (Supplementary Table 4). However, the two groups had no significant differences (*p* = 0.13, *t*-test). Therefore, the result did not support that *Ph1* solely promoted aneuploid formation in SHW-L1 and its hybrids.

### Correlation Between Aneuploid Frequency and Phenotypic Stability of RILs

To check if the aneuploidy has an effect on phenotypic uniformity, we calculated the standard deviations of several traits in 48 SHW-L1/CM32 RILs and analyzed the relationship between the standard deviations and aneuploid frequencies. We found aneuploid frequencies were positively correlated with spike length at both Wenjiang 2016 and Beijing 2017, spikelet number at Wenjiang 2017, and plant height and rate of seed-setting at Beijing 2017 (Table 3). These results indicated that aneuploidy had an important influence on phenotype uniformity of RILs although depending on specific environments.

**TABLE 3:** Correlations between aneuploid frequency and standard deviations in RILs.

Environment	Trait	Correlation coefficient	p-Value
Wenjiang 2016	Spike length	0.36*	0.01
	Spikelet number	0.18	0.20
Wenjiang 2017	Spike length	0.07	0.61
	Spikelet number	0.43**	0.002
Beijing 2017	Spike length	0.39**	0.006
	Spikelet number	0.22	0.13
	Plant height	0.54**	0.00
	Rate of seed-setting	0.35*	0.01
	Uppermost internode length	0.17	0.24

*, **Significantly correlated between aneuploid frequency and standard deviations at p = 0.05 and p = 0.01, respectively.

## Discussion

### Chromosomal Instability in Synthetic Wheat Is Associated With Univalency

Polyploids have a high tolerance to aneuploidy compared to diploids. This is well exemplified by the production of extensive series of aneuploids such as monosomics, trisomics, tetrasomics, and nullisomics in common wheat (Sears, 1954). However, the ratio of aneuploidy common wheat varieties is quite low (Riley and Kimber, 1961). In contrast, high frequencies of aneuploidy are common in newly synthesized hexaploid wheats and is progenitor-dependent (Mestiri et al., 2010; Zhang et al., 2013). Among seedlings obtained from confirmed euploid individuals of SHW-L1 there was a high frequency of aneuploid individuals indicative of chromosomal instability.

Observations on univalent behavior in this study demonstrated that the wide variation in number between plants was associated with frequent univalency in meiosis. Univalents divide abnormally in meiosis and their derivative chromosomes are often not equally transmitted to daughter nuclei or lost in the formation of micronuclei. Univalents also have a tendency to break, especially at the centromeres (Robertson, 1916; Sears, 1952). Chromosome breakage leads to production fragmented chromosomes, and the possibility of breakage-fusion causing translocations (Lukaszewski and Gustafson, 1983; Lukaszewski, 1997, 2010; Friebe et al., 2005).

It is well known that primary polyploidy is often accompanied by frequent aneuploidy (Ramsey and Schemske, 2002; Comai, 2005; Madlung et al., 2005; Feldman and Levy, 2012). However, the underlying genetic mechanism remains unknown. A QTL locus related to chromosome instability was identified from an F_2_ population derived from a cross between synthetic and natural *Arabidopsis* lines (Henry et al., 2014). While our results do not offer clear answer as to the genetic basis of high frequency of aneuploidy in synthetic wheat they do add some new information for future investigations. In common wheat and its progenitor species *T. turgidum*, *Ph1* is a key gene controlling the exclusive formation of bivalent pairing in meiosis (Okamoto, 1957; Riley and Chapman, 1958). When it is absent or inhibited, chromosomes exhibit relaxed homologous pairing and increased homoeologous pairing that is accompanied by the presence of univalents. One hypothesis for the high aneuploidy was that the *Ph1* allele in synthetic wheat was sufficiently efficient to ensure the exclusive formation of bivalents. However, that hypothesis was rejected since homoeologous pairing is not common in synthetic wheat lines (Mestiri et al., 2010; Zhang et al., 2010). The aneuploid analysis on RILs in this study also did not support the hypothesis since most of the selected euploid RILs had aneuploid frequencies over 3%. If *Ph1* is the sole genetic factor causing aneuploidy, the expected ratio should be much higher. Reciprocal cross between the synthetic and a conventional wheat line revealed no difference in aneuploidy this excluding a role of cytoplasm.

### Persistent Chromosomal Instability Affects Varietal Uniformity

The achievement of varietal uniformity is an often-encountered problem in distant hybridization breeding compared to classical breeding. Such a phenomenon is described as “long-term segregation” although the underlying genetic mechanism remains poorly understood. In this study, we observed that a high frequency of *de novo* chromosome variation persisted in euploid plants selected from an F_14_ population. We term the phenomenon persistent chromosome instability (PCI). In crosses our analyzed F_14_ lines with common wheat aneuploidy continued to influence the phenotype uniformity, showing as obvious trait differences among plants within a RIL. The phenotypic variation was correlated with the frequencies of aneuploids. These results indicated that PCI was the cause of “long-term segregation” in hybrids between synthetic and natural wheat.

### Overcoming Aneuploidy in Genetics and Breeding

The simple cross is a popular method to generate breeding and research populations such as RILs and DHs. Standard wheat varieties usually produce 1–3% aneuploid individuals (Riley and Kimber, 1961). We used 3% aneuploidy as the upper threshold to designate a chromosomally stabile line (CSL) compared to an unstabile line (CuSL). Based on these criteria, 41 out of 48 RILs were CuSL. These RILs were derived from a single-cross between the synthetic and common wheat, indicating that a single cross was not effective in eliminating the chromosomal instability. These RILs have been used in gene/QTL analysis by different groups (Yu et al., 2014; Hao et al., 2019; Yang et al., 2019). However, such analysis ignored the effects of chromosome variation on phenotyping and hence the accuracy of results could have been affected. To eliminate effects of chromosome instability, populations used in genetic analysis or breeding should retain less genomic content from the synthetic. The advanced backcross QTL approach proposed by Tanksley and Nelson (1996) provides such a way to retain favorable alleles inherited from a synthetic while reconstituting most of the genetic background to that of common wheat. This method was applied to QTL discovery in synthetic wheat (Huang et al., 2003, 2004; Liu et al., 2006). The SHW-L1 derived varieties Shumai 969 and Shumai 830 investigated in this study were chromosomally stable. They contained ∼12.5% of the genome of SHW-L1 (Hao et al., 2019). Thus, chromosome instability of nascent wheat was largely overcome by increasing the genetic background of common wheat.

## Data Availability Statement

The original contributions presented in the study are included in the article/Supplementary Material, further inquiries can be directed to the corresponding author/s.

## Author Contributions

LBZ, DL, and MH conceived and designed the study. LBZ, DX, CF, SZ, and LH performed the experiments. SN, BJ, LQZ, and ZY supervised the study. LBZ prepared the manuscript. DL and MH edited the manuscript. All authors read and approved the final manuscript.

## Conflict of Interest

The authors declare that the research was conducted in the absence of any commercial or financial relationships that could be construed as a potential conflict of interest.

## Supplementary Material

Supplementary Figure 1 FISH karyotypes: **(A)** SHW-L1/Chuanmai 32 F_1_ hybrid; **(B)** Chuanmai 32/SHW-L1 F_1_ hybrid. Green FISH signals came from probe oligo-pTa-535; red signals were from probe oligo-pSc119.2.

Supplementary Figure 2 GISH and FISH karyotypes. **(A)** SHW-L1 and Chuanmai 32; **(B)** Two seedlings from two RILs with chromosome structural variations (white arrows).

Supplementary Table 1 Chromosome composition of progenies derived from selfed euploid SHW-L1 and SHW-L1/Chuanmai 32 F_1_.

Supplementary Table 2 Type and distribution of chromosomal structural variations.

Supplementary Table 3 Number of chromosomes having structural variations.

Supplementary Table 4 Chromosome distribution and aneuploid frequency among 48 RILs from SHW-L1/CM32.
