# Supplementary figures and images for: Chromosome Stability of Synthetic-Natural Wheat Hybrids
Source: Front Plant Sci. 2021 Mar 17;12:654382. doi: 10.3389/fpls.2021.654382 (PMC8010257; doi:10.3389/fpls.2021.654382)

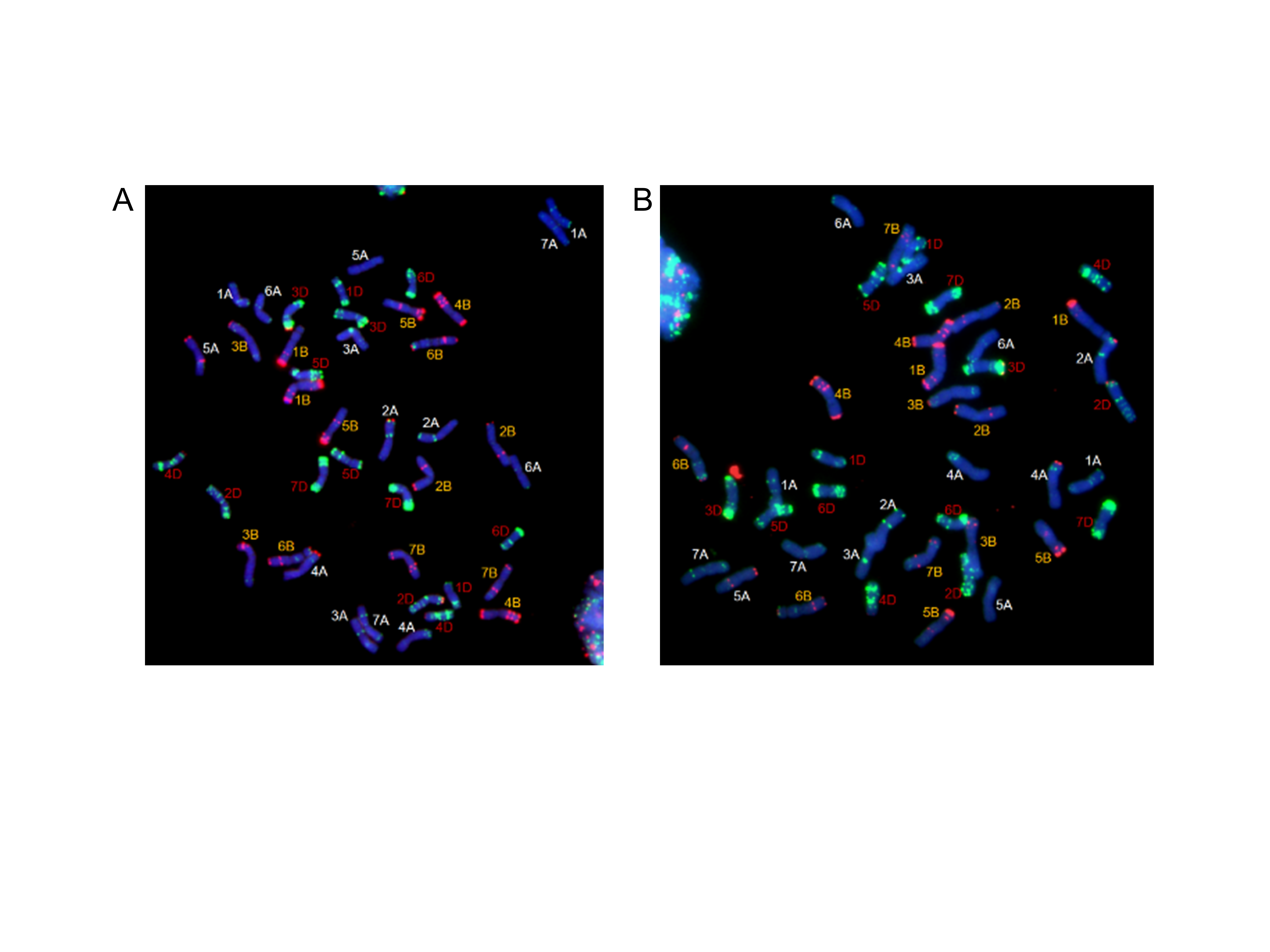

Supplement: Supplementary Figure 1 — FISH karyotypes: (A) SHW-L1/Chuanmai 32 F1 hybrid; (B) Chuanmai 32/SHW-L1 F1 hybrid. Green FISH signals came from probe oligo-pTa-535; red signals were from probe oligo-pSc119.2. [file Image_1.TIF]

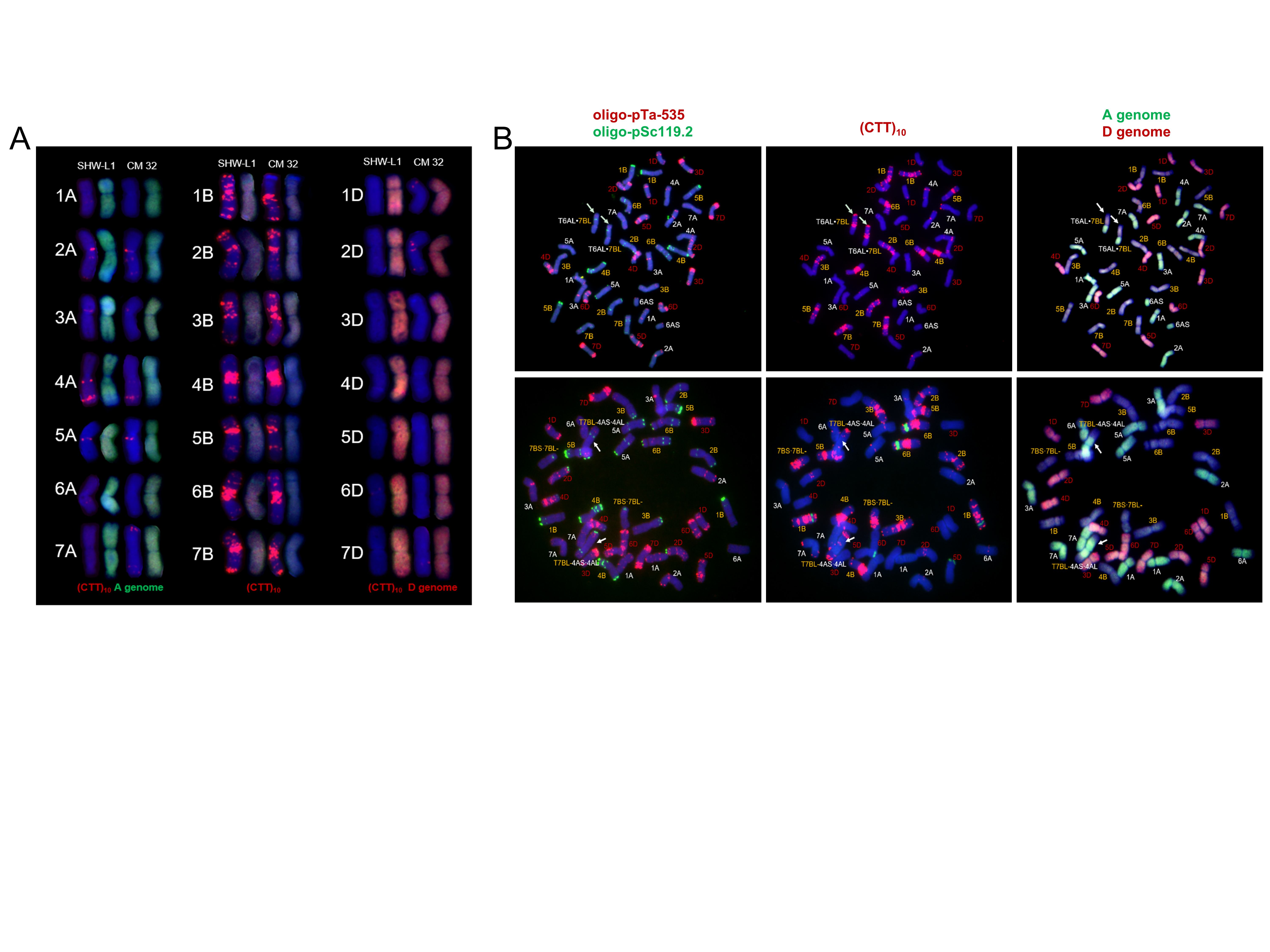

Supplement: Supplementary Figure 2 — GISH and FISH karyotypes. (A) SHW-L1 and Chuanmai 32; (B) Two seedlings from two RILs with chromosome structural variations (white arrows). [file Image_2.TIF]
