# Supplementary material for: Chromosome Stability of Synthetic-Natural Wheat Hybrids
Source: Front Plant Sci. 2021 Mar 17;12:654382. doi: 10.3389/fpls.2021.654382 (PMC8010257; doi:10.3389/fpls.2021.654382)
Supplement: Supplementary Table 3 — Number of chromosomes having structural variations. [file Table_3.DOC]

Supplementary Table 3 Number of chromosome having structural variation.

| Chromosome | SHW-L1 | SHW-L1/CM 32 F_2_ | CM32/SHW-L1 F_2_ |
| --- | --- | --- | --- |
| 1A | 0 | 2 | 2 |
| 2A | 1 | 1 | 1 |
| 3A | 0 | 0 | 1 |
| 4A | 1 | 0 | 1 |
| 5A | 1 | 4 | 2 |
| 6A | 1 | 0 | 2 |
| 7A | 0 | 2 | 2 |
| A | 4 | 9 | 11 |
| 1B | 2 | 7 | 4 |
| 2B | 2 | 4 | 1 |
| 3B | 3 | 9 | 6 |
| 4B | 6 | 3 | 4 |
| 5B | 1 | 1 | 2 |
| 6B | 4 | 1 | 8 |
| 7B | 3 | 5 | 8 |
| B | 21 | 30 | 33 |
| 1D | 1 | 2 | 1 |
| 2D | 1 | 1 | 3 |
| 3D | 1 | 7 | 3 |
| 4D | 0 | 2 | 1 |
| 5D | 0 | 1 | 4 |
| 6D | 0 | 3 | 2 |
| 7D | 1 | 4 | 3 |
| D | 4 | 20 | 17 |
| ABD | 29 | 59 | 61 |
